# Eating Behavior Traits and Success in Dietary Changes in Men with Prediabetes – the T2D-GENE Intervention

**DOI:** 10.1177/10901981251411226

**Published:** 2026-01-23

**Authors:** Noora Koivu, Maria Lankinen, Ursula Schwab

**Affiliations:** 1 University of Eastern Finland, Kuopio, Finland; 2 Kuopio University Hospital, Kuopio, Finland

**Keywords:** cognitive restraint, diet, emotional eating, intervention study, type 2 diabetes, uncontrolled eating

## Abstract

Sustained dietary change is essential for type 2 diabetes prevention, yet most Finnish men do not follow dietary recommendations. Eating behavior traits such as cognitive restraint, uncontrolled eating, and emotional eating may influence dietary success; however, their role in long-term adherence remains unclear. Moreover, men are underrepresented in eating behavior research. This study investigated whether baseline eating behavior traits predicted dietary goal achievement and changes in food intake during a 3-year lifestyle intervention among 370 older (50–75 years) men with prediabetes. The intervention promoted increased intake of vegetables, fruit, fiber, and unsaturated fats, while reducing saturated fat and sucrose. Diet was assessed with 4-day food records and eating behavior traits with the Three-Factor Eating Questionnaire-R18. Participants improved their diet quality, particularly fiber intake and fat quality. Although eating behavior traits did not predict overall success in reaching dietary goals, they were linked to specific food choices: Higher cognitive restraint predicted increased consumption of wholegrains, higher uncontrolled eating was linked to reduced intake of fatty pastries, and higher emotional eating was associated with increased nut and seed intake and decreased sugar-sweetened beverage consumption. Interestingly, traits typically associated with poorer eating habits were linked to healthier dietary changes. These results suggest that eating behavior traits may not determine overall adherence but can shape specific dietary patterns. Assessing these traits before intervention may support the design of tailored behavioral strategies. This study offers new insights into behavioral predictors of dietary change in older men with prediabetes, a group requiring personalized prevention strategies.

The global incidence of type 2 diabetes (T2D) is a major public health issue. T2D is highly preventable through lifestyle changes (International Diabetes Federation, 2021; Mayer-Davis et al., 2004; Tuomilehto et al., 2001). However, most Finnish adults, especially men, do not follow the nutritional guidelines particularly regarding fat quality and fruit and vegetable consumption (Kaartinen et al., 2020). Adherence is similarly low in other high-income Western countries (Dietary Guidelines for Americans et al., 2020; Leme et al., 2021). Given challenges in sustaining lifestyle changes and the variability in the intervention effectiveness (Bodhini et al., 2023; Hall & Kahan, 2018), identifying factors that may facilitate or hinder such changes is crucial.

Behavioral factors, such as personality, motivation and self-efficacy, have been identified as determinants of lifestyle change success (Karsten et al., 2019; Stieger et al., 2020; Teixeira et al., 2015), but the role of eating behavior traits remains unclear. Eating behavior traits illustrate motivation to eat (Bellisle, 2009; LaCaille, 2013). This study focuses on three key traits—cognitive restraint (CR, that is, conscious restriction of food intake to control body weight), uncontrolled eating (UE, that is, compulsive overeating resulting from loss of control), and emotional eating (EE, that is, managing emotions by eating)—as conceptualized in the Three-Factor Eating Questionnaire revised 18-item version (TFEQ-R18) (Karlsson et al., 2000). These are potential predictors of dietary changes, as CR is associated with healthier food choices including higher consumption of fruit, vegetables, and fish (Contento et al., 2005; de Lauzon et al., 2004; Elfhag et al., 2008; Konttinen et al., 2010; López-Cepero et al., 2021; Miller et al., 2014), while UE (Chui et al., 2020; de Lauzon et al., 2004; Keskitalo et al., 2008; Pentikäinen et al., 2018) and EE (Camilleri et al., 2014; de Lauzon et al., 2004; Keskitalo et al., 2008; Pentikäinen et al., 2018) link to higher intake of energy-dense foods.

Lifestyle intervention studies typically focus on weight loss as the success indicator, (Fielding-Singh et al., 2019; Jiskoot et al., 2022; Karsten et al., 2019; Teixeira et al., 2015), although diet quality may independently influence T2D risk (Salas-Salvadó et al., 2011, 2014). Higher baseline CR and external eating (i.e., eating in response to food presence; Van Strien et al., 1986) have predicted reduced fat and energy intake in short- and intermediate-term among women (Mason et al., 2019; Van de Laar et al., 2006). Another study found no long-term predictive value of eating behavior traits in individuals with prediabetes (Jalo et al., 2024). Future research should consider detailed food choices, as eating behavior traits may impact food selection more than macronutrient intake (Konttinen et al., 2010). Sex differences complicate this relationship: men generally score lower than women on CR, UE, and EE (Cappelleri et al., 2009; de Lauzon et al., 2004; Dohle et al., 2014; Konttinen et al., 2009; Stinson et al., 2019), and these traits may influence diet differently by sex (Camilleri et al., 2014; de Lauzon et al., 2004; Elfhag et al., 2008; Konttinen et al., 2010; Lluch et al., 2000). Older men are rarely included in eating behavior studies, yet sex-tailored strategies could improve T2D prevention (Rollo et al., 2017).

Identifying eating behavior traits that influence dietary adherence and incorporating pre-intervention screening may support personalized strategies and improve long-term outcomes in the prevention and management of T2D. Thus, we aimed to examine (1) whether baseline eating behavior traits differ between individuals who successfully achieve or maintain dietary goals and those who do not, and (2) if these traits predict changes in diet or eating behavior during a 3-year lifestyle intervention in older men with prediabetes. We hypothesize that higher baseline UE and EE will hinder dietary change success, whereas CR will support it.

## Methods

### Participants

This longitudinal study was conducted within the T2D-GENE lifestyle intervention framework, which examined the impact of low versus high genetic risk on T2D development during a group-based intervention (Lankinen et al., 2024; Schwab et al., 2021, 2023). The intervention was successful, reducing the T2D risk by 52 percent (Lankinen et al., 2024).

Participant selection for the T2D-GENE intervention has been described previously (Lankinen et al., 2024; Schwab et al., 2021, 2023). Briefly, the participants were selected from 10,197 men who had previously participated in the METSIM study (Laakso et al., 2017), randomly chosen from the population register of Kuopio, meeting inclusion criteria: (1) impaired fasting glucose (IFG) (fasting plasma glucose ≥ 5.6 mmol/l) with or without impaired glucose tolerance (IGT) (2-hour glucose 7.8–11.0 mmol/l or <7.8 mmol/l), (2) age 50–70 years, (3) body mass index (BMI) > 25 kg/m², and (4) either high or low genetic risk for T2D. Chronic diseases affecting participation were exclusion criteria.

A total of 614 participants were enrolled and received the assigned intervention (Supplemental Figure 1). The control group (*n* = 345) was excluded as they did not complete food records or eating behavior questionnaires. Intervention participants were categorized into highest (*n* = 303) or lowest tertile (*n* = 311) based on their genetic risk score, though genetic risk was not considered in our analysis. The participants, laboratory nurses and clinical nutritionists were blinded to these assignments. Data from the TFEQ-R18 at baseline and food diaries at both baseline and 3 years were available for 370 participants. None were diagnosed with T2D before the 3-year visit. These participants were included in the analysis. Baseline characteristics are shown in Table 1. The 244 dropouts were similar to included participants, except for being younger (64.4 ± 5.7 years) (FDR-*p* = .03) and having higher HbA1c (37.9 ± 3.0 mmol/mol) (FDR-*p* = .03).

**Table 1.** Baseline Characteristics in the T2D-GENE Intervention (*n* = 370), Mean + *SD* or *N* (%).

Characteristic	
Age (years)	65.7 ± 5.9
BMI (kg/m^2^)	28.5 ± 3.0
Fasting plasma glucose (mmol/l)	6.0 ± 0.3
2-hour plasma glucose (mmol/l)	6.2 ± 1.5
Blood HbA1c (mmol/mol)	37.2 ± 3.2
Education^a^	
Primary school	67 (18.7)
Secondary school	203 (56.9)
Higher level	72 (20.2)
Other	15 (4.2)
Marital status^a^	
Married/registered partnership/cohabiting	311 (87.1)
Single/divorced/widow/living apart	46 (12.9)
Responsibility of food management^b^	
With a partner	288 (80.4)
Alone	44 (12.3)
Not at all	26 (7.3)

*Note.* BMI, Body mass index; HbA1c, Hemoglobin A1c.

a *n* = 357. ^b^ *n* = 358.

This trial was approved by the Ethics Committee of the Hospital District of Northern Savo on February 9, 2016 (no. 71/2016) via an expedited review procedure and conducted in accordance with the International Declaration of Helsinki (2013). Participants gave written consent and could withdraw at any time. No compensation was provided.

### T2D-GENE Lifestyle Intervention

The 3-year T2D-GENE intervention included 5–7 group sessions, led by clinical nutritionists, to support motivation and understanding of a health-promoting lifestyle (Lankinen et al., 2024; Schwab et al., 2021, 2023; Supplemental Figure 2). Sessions focused on healthy eating, physical activity, and the role of lifestyle changes in reducing T2D risk. Participants with a BMI above 28 kg/m² received additional support through two extra sessions–also open to all–focused on healthy diet principles and eating behavior (Schwab et al., 2021, 2023). A web-based portal provided monthly educational material on diet and physical activity. Laboratory assessments were conducted at baseline, at 1, 2, and 3 years.

### Assessment of Baseline Eating Behavior Traits

The TFEQ-R18 (Karlsson et al., 2000) was used to assess eating behavior traits: CR, UE, and EE (Supplemental Text 1) at baseline, at 0.5, 1, 2, and 3 years (Supplemental Figure 2). For each trait, the score ranges from 0 to 100, with higher scores indicating greater intensity. TFEQ-R18 has been validated in individuals with and without obesity (de Lauzon et al., 2004; Elfhag & Linné, 2005; Karlsson et al., 2000). The Finnish version, used in this study, was translated and back-translated from the original version by the Finnish Association for the Study of Obesity and has shown good structural validity in young Finnish females with varying BMI (Anglé et al., 2009).

Although the T2D-GENE intervention did not specifically target eating behavior changes, two additional lecture-based sessions for participants with BMI over 28 kg/m² addressed diet quality, food energy density, and strategies for managing overeating, including mindful eating, flexible restraint, craving management, and practical recommendations such as identifying overeating triggers and planning grocery shopping (Schwab et al., 2021, 2023).

A previous study from this cohort (Malkki-Keinänen et al., 2022) demonstrated that a modified 15-item TFEQ could substitute R18. However, the TFEQ-R18 remains the most consistent with prior studies. Both versions were used in the analysis; nonetheless, only TFEQ-R18 results are reported, as the findings were highly similar.

### Assessment of Diet

Dietary guidance and goals during the intervention were based on the Nordic and Finnish nutrition recommendations (Nordic Council of Ministers, 2014). The target nutrient composition of the T2D-GENE diet is presented in Table 2 (Schwab et al., 2021, 2023).

**Table 2.** Dietary Goals in the T2D-GENE Intervention.

Dietary variable	Goal
Vegetables, fruit, and berries	≥500 g/day
Carbohydrates	45–60 E%
Fiber	≥35 g/day
Sucrose	<10 E%
Fat	25–40 E%
Saturated fat	<10 E%
Monounsaturated and polyunsaturated fat Polyunsaturated fat	≥2/3 of total fat 5–10 E%
Monounsaturated fat	10–20 E%
Omega-3 fatty acids	≥1 E%

*Note.* E%, percent of energy intake.

Dietary intake was assessed using 4-day food records, including one weekend day, at baseline and 0.5, 1, 2, and 3 years (Schwab et al., 2021; Supplemental Figure 2). The participants were instructed to report all foods and beverages consumed, using food weighing or portion size estimates for accuracy. Clinical nutritionists reviewed the records, and dietary intake was calculated using AivoDiet software version 2.2.0.0 (Mashie FoodTech Solutions Finland Oy, Turku, Finland). Participants received written feedback and individualized dietary guidance at each time point.

The average daily intake of total energy (kcal), fiber (g), vitamin C (mg), folate (µg), and the percentages of the energy (E%) obtained from protein, carbohydrates, fat, monounsaturated fatty acids, polyunsaturated fatty acids, saturated fatty acids, omega-3 fatty acids and sucrose were analyzed. The consumption of foods was reported as grams per day. The foods reported in the food records were grouped into 11 food groups to evaluate overall dietary patterns (Supplemental Table 1). The groups were based on key nutritional principles aimed at preventing and treating T2D (Aas et al., 2023). Both food groups and individual foods, including those outside the groups, were analyzed. Results for variables deemed relevant were reported.

### Statistical Methods

Baseline characteristics are presented as means ± SDs and percentages. Differences between participants and dropouts were assessed using the Mann–Whitney U test and chi-square test. The baseline eating behavior variables and BMI did not exhibit a normal distribution; their results are reported as means ± SDs.

Food intake variables are presented as means ± 95% CIs at baseline and 3 years. Most did not follow normal distribution. Delta values (baseline to 3-year changes) were calculated for dietary variables and eating behavior traits. Delta values followed a normal distribution. To assess changes in food intake, BMI, and eating behavior traits from baseline to 3 years, we used the Wilcoxon signed-rank test for non-parametric data and the paired *t*-test for parametric data.

We compared baseline eating behavior traits between participants who did or did not meet dietary goals at 3 years. The Mann–Whitney U test compared two groups (goal achieved vs. not achieved), while the Kruskal–Wallis test compared three groups (under, not achieved, and exceeded goal).

Spearman’s correlation analysis was conducted to assess correlations between baseline BMI and eating behavior traits, and to screen for potential associations between baseline eating behavior traits and changes in dietary variables (nutrients, food groups and individual foods). Delta values of dietary factors significantly correlated with baseline eating behavior traits were further analyzed using linear regression models, using eating behavior traits as predictors and dietary changes as outcomes. Baseline BMI was included as a covariate in the second step to assess its influence on these relationships. Assumptions for linear regression were met: the residuals of all models were normally distributed and linear, no autocorrelation, and no multicollinearity (VIF values < 5).

Multiple comparisons were controlled using the Benjamini-Hochberg (FDR) method. FDR-adjusted *p*-values were used throughout, except for correlations between BMI and baseline eating behavior traits, and in exploratory correlations between baseline eating behavior traits and diet (Supplemental Table 2). A total of 8 comparisons for baseline characteristics, 14 for foods, 12 for nutrients, 3 for eating behavior traits, and 3 for linear regression models between changes in eating behavior traits and baseline eating behavior traits, 15 for linear regression models between foods and eating behavior traits, 6 for linear regression models between nutrients and eating behavior traits, and 30 achieving dietary goals were tested. Significance was set at FDR-*p* < .05, and *p* < .05 for exploratory analyses and correlations between BMI and eating behavior. All analyses were conducted using SPSS version 27.0 (IBM Inc., Armonk, NY) except for FDR corrections in R version 4.3.1 (R Core Team, 2023).

## Results

### Baseline Eating Behavior Traits and Body Mass Index

CR had the highest baseline score (mean 50.3 ± 15.6), followed by UE (mean 26.9 ± 15.7) and EE (mean 18.7 ± 20.4).

A detailed analysis of eating behavior changes during the intervention will be reported elsewhere. Briefly, the data set, which included both baseline and year 3 data, showed that during the T2D-GENE intervention, participants improved their CR scores, while UE and EE scores decreased. Two participants were excluded due to incomplete data.

Baseline BMI correlated positively with baseline UE (*r* = 0.20; *p* < .001) and EE (*r* = .17; *p* < .001). BMI change over 3 years was not significant.

### Changes in Dietary Variables During the Intervention

**Figure 1.**
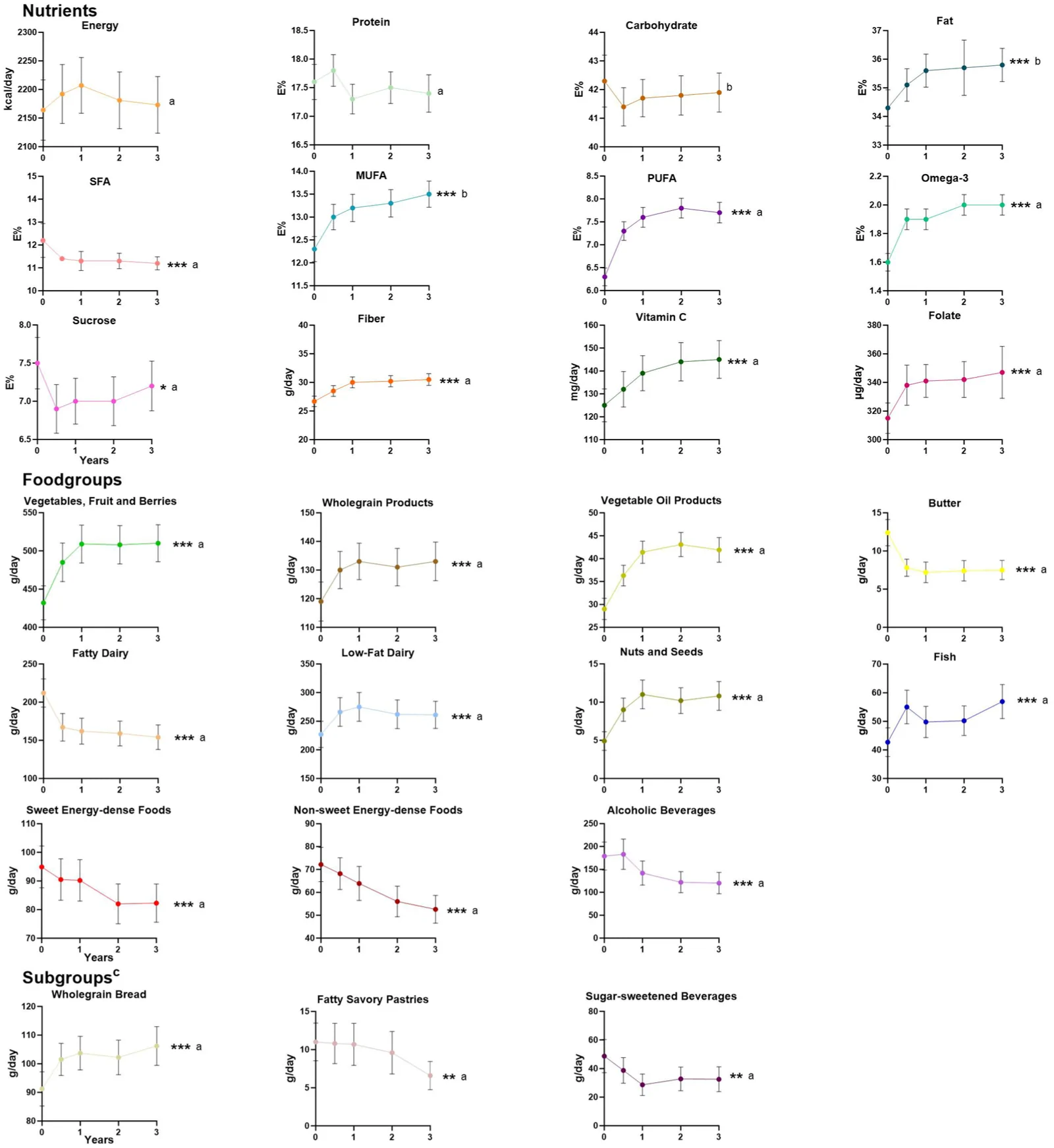
Changes in the Intake of Nutrients, Food Groups, and Subgroups During the 3-Year Intervention. *Note.* E%, energy percent; SFA, saturated fatty acids; MUFA, monounsaturated fatty acids; PUFA, polyunsaturated fatty acids. T_0_ *n* = 370; T_0.5_ *n* = 364; T_1_ *n* = 362; T_2_ *n* = 365; T_3_ *n* = 370 The change in nutrient intake was assessed using ^a^ Wilcoxon signed rank test or ^b^ paired *t*-test for the 0- to 3-year difference. ^c^ Subgroups include individual foods within or outside food groups; only those with significant associations in regression analyses are shown. FDR-*p* <.05*, FDR-*p* < .01**, FDR-*p* <.001***

During the intervention, the intake of dietary fat (FDR-*p* < .001), saturated fatty acids (FDR-*p* < .001), and sucrose (FDR-*p* = .02) decreased significantly, while the intake of monounsaturated and polyunsaturated fatty acids, omega-3 fatty acids, fiber, vitamin C and folate increased significantly (FDR-*p* < .001 for all) (Figure 1). The intake of energy (FDR-*p* = .60), protein (FDR-*p* = .16) and carbohydrates (FDR-*p* = .19) did not change significantly.

The intake of vegetables, fruit, and berries, wholegrain products, vegetable oil products, nuts and seeds, fish and low-fat dairy products increased significantly (FDR-*p* < .001 for all) during the intervention, while the intake of butter, fatty dairy products, sweet and non-sweet energy-dense foods and alcoholic beverages decreased significantly (FDR-*p* < .001 for all) (Figure 1). Changes in both grouped and ungrouped individual food items (i.e., subgroups) are also shown in Figure 1. We only present the foods that were significant in linear regression analysis (Table 4). The intake of wholegrain bread (FDR-*p* < .001) increased, while the intake of sugar-sweetened beverages (FDR-*p* = .009), and fatty savory pastries (FDR-*p* = .003) decreased during the intervention.

### Associations Between Baseline Eating Behavior Traits and Change in Eating Behavior Traits During the Intervention

Eating behavior scores at baseline predicted changes in eating behavior traits during the intervention (Supplemental Table 3). Higher baseline CR and UE scores were associated with decreases in CR and UE during the intervention (FDR-*p* < .001). Higher baseline EE scores were linked to increases in CR and UE, while also being associated with a decrease in EE scores (FDR-*p* < .001). Baseline BMI did not influence these associations.

### The Association Between Baseline Eating Behavior Traits and Adherence to Nutritional Goals of the Intervention

The differences in eating behavior traits at baseline between participants who achieved or remained the nutritional goals (defined in Table 2) during the three-year lifestyle intervention and who did not are presented in Table 3. After the FDR corrections, none of the associations were significant.

**Table 3.** Baseline Eating Behavior Traits in Goal Achievers and Non-Achievers.

Nutritional variable	Eating behavior trait	Intake at 3 years, *n* (%)	Mean rank	*p*-value	Adjusted *p*-value ^c^
Vegetables, fruit, and berries	T_0_ CR	<500 g/day *n* = 189 (51)	179.77	.29^a^	.52
		>500 g/day *n* = 181 (49)	191.48		
	T_0_ UE	<500 g/day *n* = 189 (51)	182.80	.62^a^	.74
		>500 g/day *n* = 181 (49)	188.81		
	T_0_ EE	<500 g/day *n* = 189 (51)	170,63	.005*^a^	.15
		>500 g/day *n* = 181 (49)	201.02		
Carbohydrates	T_0_ CR	<45 E% *n* = 255 (68.9)	186.11	.89^b^	.89
		45–60 E% *n* = 114 (30.8)	183.73		
		>60 E% *n* = 1 (0.3)	232.00		
	T_0_ UE	<45 E% *n* = 255 (68.9)	190.81	.36^b^	.539
		45–60 E% *n* = 114 (30.8)	173.61		
		>60 E% *n* = 1 (0.3)	188.00		
	T_0_ EE	<45 E% *n* = 255 (68.9)	192.81	.08^b^	.31
		45–60 E% *n* = 114 (30.8)	170.13		
		>60 E% *n* = 1 (0.3)	73.00		
Sucrose	T_0_ CR	<10 E% *n* = 300 (81)	185.97	.86^a^	.89
		>10 E% *n* = 70 (19)	183.51		
	T_0_ UE	<10 E% *n* = 300 (81)	191.04	.04*^a^	.22
		>10 E% *n* = 70 (19)	161.77		
	T_0_ EE	<10 E% *n* = 300 (81)	190.71	.04*^a^	.22
		>10 E% *n* = 70 (19)	163.19		
Fiber	T_0_ CR	< 35 g/day *n* = 271 (73)	182.01	.29^a^	.52
		> 35 g/day *n* = 99 (27)	195.07		
	T_0_ UE	< 35 g/day *n* = 271 (73)	181.86	.28^a^	.52
		> 35 g/day *n* = 99 (27)	195.45		
	T_0_ EE	< 35 g/day *n* = 271 (73)	178.86	.04*^a^	.22
		> 35 g/day *n* = 99 (27)	204.17		
Fat	T_0_ CR	<25 E% *n* = 9 (2)	128.5	.21^b^	.52
		25–40 E% *n* = 281 (76)	184.85		
		>40 E% *n* = 80 (22)	194.19		
	T_0_ UE	<25 E% *n* = 9 (2)	239.50	.32^b^	.53
		25–40 E% *n* = 281 (76)	184.16		
		>40 E% *n* = 80 (22)	184.24		
	T_0_ EE	<25 E% *n* = 9 (2)	175.00	.11^b^	.373
		25–40 E% *n* = 281 (76)	179.77		
		>40 E% *n* = 80 (22)	206.80		
Saturated fat	T_0_ CR	<10 E% *n* = 129 (35)	190.55	.50^a^	.66
		> 10 E% *n* = 241 (65)	182.80		
	T_0_ UE	<10 E% *n* = 129 (35)	196.85	.13^a^	.40
		> 10 E% *n* = 241 (65)	179.43		
	T_0_ EE	<10 E% *n* = 129 (35)	201.39	.03*^a^	.22
		> 10 E% *n* = 241 (65)	177.0		
Monounsaturated fat and polyunsaturated fat	T_0_ CR	<2/3 of total fat *n* = 324 (88)	185.19	.88^a^	.89
		>2/3 of total fat *n* = 46 (12)	187.66		
	T_0_ UE	<2/3 of total fat *n* = 324 (88)	183.55	.35^a^	.54
		>2/3 of total fat *n* = 46 (12)	199.21		
	T_0_ EE	<2/3 of total fat *n* = 324 (88)	183.11	.24^a^	.52
		>2/3 of total fat *n* = 46 (12)	202.35		
Polyunsaturated fat	T_0_ CR	<5 E% *n* = 35 (9)	151.49	.06^b^	.25
		5–10 E% *n* = 292 (79)	186.14		
		>10 E% *n* = 43 (12)	208.84		
	T_0_ UE	<5 E% *n* = 35 (9)	184.01	.42^b^	.60
		5–10 E% *n* = 292 (79)	174.86		
		>10 E% *n* = 43 (12)	204.27		
	T_0_ EE	<5 E% *n* = 35 (9)	150.86	.04*^b^	.22
		5–10 E% *n* = 292 (79)	185.93		
		>10 E% *n* = 43 (12)	210.77		
Monounsaturated fat	T_0_ CR	<10 E% *n* = 31 (8)	173.95	.60^b^	.74
		10–20 E% *n* = 333 (90)	185.94		
		>20 E% *n* = 6 (2)	220.70		
	T_0_ UE	<10 E% *n* = 31 (8)	199.82	.69^b^	.79
		10–20 E% *n* = 333 (90)	183.90		
		>20 E% *n* = 6 (2)	200.17		
	T_0_ EE	<10 E% *n* = 31 (8)	158.50	.19^b^	.52
		10–20 E% *n* = 333 (90)	187.23		
		>20 E% *n* = 6 (2)	229.25		
Omega-3	T_0_ CR	<1 E% *n* = 19 (5)	179.55	.80^a^	.89
		>1 E% *n* = 351 (95)	185.82		
	T_0_ UE	<1 E% *n* = 19 (5)	167.84	.46^a^	.63
		>1 E% *n* = 351 (95)	186.46		
	T_0_ EE	<1 E% *n* = 19 (5)	161.39	.29^a^	.52
		>1 E% *n* = 351 (95)	186.80		

*Note.* CR, Cognitive restraint; E%, percent of daily energy intake; EE, Emotional eating; T_0_, Baseline value; UE, Uncontrolled eating.

a Mann–Whitney U test. ^b^ Kruskal–Wallis test. ^c^ Benjamini–Hochberg False Discovery Rate correction.

FDR-*p* <.05*, FDR-*p* <.01**, FDR-*p* <.001***

### Associations Between Baseline Eating Behavior Traits and Changes in Dietary Variables

Baseline CR was directly associated with change in the wholegrain products (FDR-*p* = .04), and wholegrain bread (FDR-*p* = .04) (Table 4). Baseline UE was inversely associated with change in the intake of fatty savory pastries (FDR-*p* = .03). Baseline EE was inversely associated with the intake of sugar-sweetened beverages (FDR-*p* = .01) and directly with change in the intake of nuts and seeds (FDR-*p* = .04). Baseline BMI moderated the associations between CR and wholegrain intake, as well as EE and nut and seed consumption, with these associations becoming non-significant after adjusting for BMI.

**Table 4.** Baseline Eating Behavior Traits Predicting Changes in Dietary Variables^a^ in the T2D-GENE Intervention (*n* = 370): A Linear Regression Analysis.

Dietary change	Predictor	R	Adjusted *R*^2^	b	*SE* (b)	Standardized beta	t	*p*-value	Adjusted *p*-value ^b^
**Nutrients**									
Δ Fiber g/day		0.18	0.03						
	T_0_ CR			0.07	0.03	0.12	2.33	.02*	.05
	T_0_UE			0.07	0.04	0.12	1.95	.05	.09
	T_0_EE			0.02	0.03	0.04	0.67	.50	.50
Δ SFA E%		0.15	0.02						
	T_0_ CR			0.02	0.01	0.12	2.20	.03*	.05
**Food groups and subgroups**									
Δ Wholegrain products		0.12	0.01						
	T_0_ CR			0.54	0.24	0.12	2.29	.02*	.04*
Δ Wholegrain bread		0.13	0.01						
	T_0_ CR			0.55	0.23	0.13	2.41	.02*	.04*
Δ Nuts and seeds		.12	0.01						
	T_0_ EE			0.11	0.05	0.12	2.40	.02*	.04*
Δ Sugar-sweetened beverages		0.16	0.02						
	T_0_ UE			–0.89	0.29	-0.16	–3.10	.002**	.01*
Δ Fatty savory pastries		0.17	0.02						
	T_0_CR			0.15	0.09	0.09	1.63	.10	.11
	T_0_ UE			–0.24	0.09	-0.13	–2.57	.01*	.03*

*Note.* Δ, delta value, that is, change from 0 to 3 years; CR, Cognitive restraint; E%, percent of daily energy intake; EE, Emotional eating; SFA, saturated fatty acids; T_0_, baseline value; UE, Uncontrolled eating.

a Dietary factors that showed significant correlations with baseline eating behavior traits were included in the linear regression models. The significant and relevant associations from these models are presented in this table. All significant correlations are provided in Supplemental Table 2. ^b^ Benjamini–Hochberg False Discovery Rate correction.

FDR-p < .05*, FDR-p < .01**, FDR-p < .001***.

## Discussion

This study examined the relationship between baseline eating behavior traits and long-term dietary changes in older men with prediabetes. Diet quality improved, and dietary goals were largely achieved or maintained while total energy intake remained stable during the intervention. The dietary changes, particularly the increase in fiber and healthier fats, and the reduction in saturated fats, may enhance insulin sensitivity and aid in T2D prevention (Aas et al., 2023; Thomas & Pfeiffer, 2012). Our findings are consistent with previous lifestyle interventions in middle-aged individuals with prediabetes or T2D, emphasizing the potential of dietary improvements in managing diabetes risk (Mayer-Davis et al., 2004; Tuomilehto et al., 2001). Contrary to our hypothesis, baseline eating behavior traits did not differ significantly between participants who achieved or maintained dietary goals and those who did not, but they predicted long-term dietary changes.

Baseline CR score was notably higher than UE and EE, contrasting with previous studies that included younger participants and either both sexes (Keränen et al., 2009; Nevanperä et al., 2015; Nurkkala et al., 2015) or women (Mason et al., 2019). Older individuals typically exhibit higher CR and lower overeating tendencies than younger (Keskitalo et al., 2008; Konttinen et al., 2010; Löffler et al., 2015; van Strien et al., 2009). In addition, men tend to score lower in CR, UE, and EE than women (Cappelleri et al., 2009; de Lauzon et al., 2004; Dohle et al., 2014; Konttinen et al., 2009; Stinson et al., 2019). The baseline BMI in our study was lower than in previous studies (Keränen et al., 2009; Nevanperä et al., 2015; Nurkkala et al., 2015), and, as in our study, BMI has been directly associated with UE and EE (Alqahtani & Alhazmi, 2025; Mason et al., 2019).

The lack of significant differences between baseline eating behavior traits and achieving dietary goals may reflect lower relevance of EE and UE in older men (Keränen et al., 2009; Nevanperä et al., 2015; Nurkkala et al., 2015). Alternatively, the assessment of the traits may not have been fully suitable for this population. However, the results were similar with the TFEQ-R15, which was a specifically adapted version for this population (Malkki-Keinänen et al., 2022). Another possible explanation is that the smaller sample sizes in some groups may have reduced statistical power, limiting our ability to detect potential differences. In addition, other behavioral factors are known to influence lifestyle changes (Karsten et al., 2019; Stieger et al., 2020; Teixeira et al., 2015) suggesting that screening for baseline eating behavior traits alone may not be sufficient to predict adherence to dietary goals. The intervention’s nutrient-focused goals may further explain the lack of association, as eating behavior traits may relate more strongly to food choices than nutrient intake (Konttinen et al., 2010), a pattern supported by our finding that baseline traits predicted changes in specific food consumption rather than nutrients.

As hypothesized, a higher CR score at baseline predicted health-promoting dietary changes, namely increased wholegrain intake, supporting earlier cross-sectional findings (Borg et al., 2004; Goulet et al., 2008; Keränen et al., 2011). Our results extend prior research by suggesting that CR may predict specific dietary improvements longitudinally, particularly increased wholegrain consumption in older men with prediabetes. Despite modest associations, CR may support T2D prevention in men with prediabetes, given the protective role of wholegrain-rich diets (Hu et al., 2020). Further studies are needed to establish its predictive value in long-term lifestyle interventions.

Contrary to our hypothesis, higher baseline UE and EE predicted favorable dietary changes, which is unexpected, given their usual association with poorer diet quality (Camilleri et al., 2014; Chui et al., 2020; de Lauzon et al., 2004; Keskitalo et al., 2008; Pentikäinen et al., 2018). Although these traits have not previously been shown to predict dietary outcomes, our findings are in line with Van de Laar et al. (2006), who reported that external eating, behaviorally similar to UE, predicted reduced energy intake in women. UE and related traits have also been linked to positive outcomes in weight loss interventions (Bryant et al., 2012; Karsten et al., 2019; McLoughlin et al., 2017), suggesting they may influence energy balance. One explanation for these counterintuitive findings is that individuals with higher baseline UE and EE may have experienced greater reductions in these behaviors during the intervention, facilitating dietary improvements, as previous research has associated reduced overeating behaviors with improvements in diet (Miller et al., 2014; Van Strien & Van de Laar, 2008). Thus, focusing on reducing UE and EE could enhance dietary changes. During our intervention, overeating behaviors were addressed in two additional group sessions for participants with a BMI over 28 kg/m^2^, covering topics like mindful eating, flexible restraint, and identifying overeating triggers. While this aspect of the intervention may have been beneficial for individuals with higher baseline UE and EE, broader integration of these themes might have yielded greater benefits. Although we did not evaluate the effectiveness of these strategies due to the absence of a control group, such practical approaches could improve the applicability of similar interventions in practice. Furthermore, UE and EE have been shown to decrease during interventions with (Miller et al., 2014; Nevanperä et al., 2015) and without specifically targeting eating behaviors (Delahanty et al., 2013; Salmela et al., 2023). Although both are overeating behaviors, UE and EE predicted different dietary changes, suggesting that tailored approaches may be needed. Future studies should determine the most effective methods for diverse individuals.

### Strengths and Limitations

Our study’s strengths include the focus on older men, an underrepresented population in eating behavior research. Despite this, the results are limited by the participants’ prediabetic status, overweight or obesity, and their male sex. However, this group particularly needs effective T2D prevention strategies. Participant numbers remained high, even though some food records and TFEQ-R18 questionnaires were not returned, limiting the statistical power and ability to detect all potential changes.

Compared with previous studies, our study provides a more detailed analysis by examining both nutrient intake and food consumption changes. However, expected stronger associations between eating behavior traits and dietary changes were not observed, possibly due to participants’ already better diet quality compared to the average Finnish population (Kaartinen et al., 2020). Furthermore, the participants may have been more health-conscious and motivated to make changes due to their prediabetes awareness.

The longitudinal design allowed us to track changes in diet and eating behavior traits over time. However, the intervention effect could not be confirmed, as neither food records nor eating behavior traits were assessed in the control group. Maintaining food records for at least 3 days, including a weekend day is regarded as representative of usual intake (Gibney, 2009). While food records provide a more accurate representation of actual intake compared to food frequency questionnaires, they rely on self-reporting, which could introduce inaccuracies. To minimize this, participants were guided to use household measures and provided with portion size references. While underreporting is a concern, it is more common among women and individuals with obesity and may not have significantly affected our study (Baranowski, 2013; Freedman et al., 2014; Hirvonen et al., 1997; Poslusna et al., 2009).

To assess eating behavior traits, we used TFEQ-R18, which is validated in various populations with and without obesity (de Lauzon et al., 2004; Elfhag & Linné, 2005; Karlsson et al., 2000). Other questionnaires exist that measure the same phenomena but are not fully comparable. Broader questionnaires, such as the original TFEQ, can be burdensome for participants. TFEQ-R18, being widely used, brief, and user-friendly, was therefore a strong choice for this study.

### Implications for Research and Practice

Our study presents novel findings, suggesting that baseline eating behavior traits may serve as predictors for specific food choices among older men with prediabetes, although they may not be key factors for long-term adherence to dietary goals. Overeating tendencies were more associated with reductions in unhealthy food intake, while CR was linked to increasing healthy foods. Men with higher baseline UE and EE may benefit from more intensive interventions that emphasize behavioral factors. Health care professionals working with men with prediabetes should consider baseline eating behavior traits when designing interventions. Early assessment of eating behavior traits could help personalize interventions, enhancing their long-term effectiveness. Future research should explore whether tailored interventions are needed for different types of eating behaviors and identify the most effective methods for each. Moreover, it is essential to determine whether other behavioral characteristics, beyond eating behaviors, should be incorporated into dietary interventions to create a more comprehensive approach to preventing and managing T2D.

## Supplemental Material

sj-docx-1-heb-10.1177_10901981251411226 – Supplemental material for Eating Behavior Traits and Success in Dietary Changes in Men with Prediabetes – the T2D-GENE Intervention Supplemental material, sj-docx-1-heb-10.1177_10901981251411226 for Eating Behavior Traits and Success in Dietary Changes in Men with Prediabetes – the T2D-GENE Intervention by Noora Koivu, Maria Lankinen and Ursula Schwab in Health Education & Behavior

sj-docx-2-heb-10.1177_10901981251411226 – Supplemental material for Eating Behavior Traits and Success in Dietary Changes in Men with Prediabetes – the T2D-GENE Intervention Supplemental material, sj-docx-2-heb-10.1177_10901981251411226 for Eating Behavior Traits and Success in Dietary Changes in Men with Prediabetes – the T2D-GENE Intervention by Noora Koivu, Maria Lankinen and Ursula Schwab in Health Education & Behavior

sj-docx-3-heb-10.1177_10901981251411226 – Supplemental material for Eating Behavior Traits and Success in Dietary Changes in Men with Prediabetes – the T2D-GENE Intervention Supplemental material, sj-docx-3-heb-10.1177_10901981251411226 for Eating Behavior Traits and Success in Dietary Changes in Men with Prediabetes – the T2D-GENE Intervention by Noora Koivu, Maria Lankinen and Ursula Schwab in Health Education & Behavior

sj-docx-4-heb-10.1177_10901981251411226 – Supplemental material for Eating Behavior Traits and Success in Dietary Changes in Men with Prediabetes – the T2D-GENE Intervention Supplemental material, sj-docx-4-heb-10.1177_10901981251411226 for Eating Behavior Traits and Success in Dietary Changes in Men with Prediabetes – the T2D-GENE Intervention by Noora Koivu, Maria Lankinen and Ursula Schwab in Health Education & Behavior

sj-docx-5-heb-10.1177_10901981251411226 – Supplemental material for Eating Behavior Traits and Success in Dietary Changes in Men with Prediabetes – the T2D-GENE Intervention Supplemental material, sj-docx-5-heb-10.1177_10901981251411226 for Eating Behavior Traits and Success in Dietary Changes in Men with Prediabetes – the T2D-GENE Intervention by Noora Koivu, Maria Lankinen and Ursula Schwab in Health Education & Behavior

sj-docx-6-heb-10.1177_10901981251411226 – Supplemental material for Eating Behavior Traits and Success in Dietary Changes in Men with Prediabetes – the T2D-GENE Intervention Supplemental material, sj-docx-6-heb-10.1177_10901981251411226 for Eating Behavior Traits and Success in Dietary Changes in Men with Prediabetes – the T2D-GENE Intervention by Noora Koivu, Maria Lankinen and Ursula Schwab in Health Education & Behavior
