# Supplementary material for: Eating Behavior Traits and Success in Dietary Changes in Men with Prediabetes – the T2D-GENE Intervention
Source: Health Educ Behav. 2026 Jan 23;53(5):440–51. doi: 10.1177/10901981251411226 (PMC13546412; doi:10.1177/10901981251411226)
Supplement: Supplementary file 1 [file sj-docx-1-heb-10.1177_10901981251411226.docx]

Suitable participants invited from the METSIM study (n=1558)

Excluded (n=930)

- Not meeting the inclusion criteria (n=37)
- Declined to participate (n=548)
- Allocated to control group (n=345)

Eligible participants (n=**370**)

T2D-GENE INTERVENTION (n=628)

Did not receive allocated intervention (n=14)

Lost to follow up (n=65)

- Discontinued intervention (n=39)
  - Disease (n=15)
  - Lack of motivation (n=7)
  - Reason not known (n=8)
  - Death (n=4)
  - Moved to another country/city (n=2)
  - Family reasons (n=1)
  - Busy (n=2)
- Diabetes cases at 1 or 2 years (n=26)

Did not have TFEQ-R18 at baseline and 4-day food records both at baseline and at the end of the intervention available (n=157)

Completed the T2D-GENE intervention (n=549)

- including diabetes cases at 3-year visit (n=22)

HIGH GENETIC RISK GROUP (n=313)

ENROLLMENT

LOW GENETIC RISK GROUP (n=315)
