# Supplementary material for: Eating Behavior Traits and Success in Dietary Changes in Men with Prediabetes – the T2D-GENE Intervention
Source: Health Educ Behav. 2026 Jan 23;53(5):440–51. doi: 10.1177/10901981251411226 (PMC13546412; doi:10.1177/10901981251411226)
Supplement: Supplementary file 2 [file sj-docx-2-heb-10.1177_10901981251411226.docx]

Supplementary Figure 2. Study Design of the T2D-GENE intervention. FR, Food Record; TFEQ-R18, The Three-Factor Eating Questionnaire Revised 18-item
