# Supplementary material for: Eating Behavior Traits and Success in Dietary Changes in Men with Prediabetes – the T2D-GENE Intervention
Source: Health Educ Behav. 2026 Jan 23;53(5):440–51. doi: 10.1177/10901981251411226 (PMC13546412; doi:10.1177/10901981251411226)
Supplement: Supplementary file 3 [file sj-docx-3-heb-10.1177_10901981251411226.docx]

Supplementary Text 1. Detailed Description of the Three-Factor Eating Questionnaire (TFEQ-R18)

The Three-Factor Eating Questionnaire Revised 18-item (TFEQ-R18) (Karlsson et al., 2000) was used to assess the intensity of three eating behavior traits: (1) Cognitive restraint, CR (2) Uncontrolled eating, UE and (3) Emotional eating, EE. TFEQ-R18 is a shortened, revised version of the original 51-item TFEQ (Stunkard, 1985). TFEQ-R18 contains 18 questions: 6 items assessing CR (e.g., “I deliberately take small helpings as a means of controlling my weight.”), 9 assessing UE (e.g., “When I see a real delicacy, I often get so hungry that I have to eat right away.”) and 3 assessing EE (e.g., “When I feel lonely, I console myself by eating.”). Respondents provide answers on a 4-point Likert-scale, yielding scores between 1 and 4 for each item, whit one item rated on an 8-point Likert-scale. To ensure compatibility, scores are rescaled to 0-100 range for each eating behavior trait using the following formula: [(raw score – minimum raw score)/possible raw score range *100] (de Lauzon et al., 2004). Higher scores indicate greater intensity of a specific eating behavior trait.

References

de Lauzon, B. de, Romon, M., Deschamps, V., Lafay, L., Borys, J. M., Karlsson, J., Ducimetiere, P., & Charles, M. A. (2004). Three-Factor Eating Questionnaire-R18 is able to distinguish among different eating patterns in a general population. *The Journal of Nutrition*, *134*(9), 2372–2380. https://doi.org/10.1093/jn/134.9.2372

Karlsson, J., Persson, L.-O., Sjöström, L., & Sullivan, M. (2000). Psychometric properties and factor structure of the Three-Factor Eating Questionnaire (TFEQ) in obese men and women. Results from the Swedish Obese Subjects (SOS) study. *INTERNATIONAL JOURNAL OF OBESITY*, *24*(12), 1715–1725. https://doi.org/10.1038/sj.ijo.0801442

Stunkard AJ, Messick S (1985). The three-factor eating questionnaire to measure dietary restraint, disinhibition and hunger. *J Psychosom Res*. 29(1):71-83. doi:10.1016/0022-3999(85)90010-8
