# Supplementary material for: Eating Behavior Traits and Success in Dietary Changes in Men with Prediabetes – the T2D-GENE Intervention
Source: Health Educ Behav. 2026 Jan 23;53(5):440–51. doi: 10.1177/10901981251411226 (PMC13546412; doi:10.1177/10901981251411226)
Supplement: Supplementary file 4 [file sj-docx-4-heb-10.1177_10901981251411226.docx]

Supplementary Table 1

*Categorization of Food Items From 4-day Food Records in the T2D-GENE Intervention*

| Food Group | Foods Included in the Food Group |
| --- | --- |
| Low-fat Dairy | low-fat fermented dairy products (fat <1%), low-fat cheese (fat≤17%), skimmed milk, vegetable oil cheeses, vegetable oil-based creams (fat<15%), low-fat creams (fat<10%) |
| Fatty Dairy | normal-fat milk (fat 1-1.5%), fatty milk (fat>1.5%), fatty fermented dairy products (fat>1%), fatty cheeses (fat>17%), fatty creams(fat>10%) |
| Vegetables, Fruits and Berries | root vegetables, vegetables, legumes, mushrooms, fruit, berries |
| Whole-grain Products | whole-grain breads (fiber≥ 6%), crispbreads (fiber≥ 10%), whole-grain snack bars (fiber≥ 6%), whole-grain flour and other whole-grain products (fiber≥ 6%), whole-grain porridges, whole-grain cereals (fiber≥ 6%) |
| Butter | butter and butter-based spreads |
| Vegetable Oil Products | vegetable oils, vegetable oil-based spreads, liquid margarines |
| Fish | fresh, cooked and smoked fish |
| Non-sweet Energy-dense Foods | salty fatty pastries, mayonnaise, sausages, salty snacks (e.g. salted peanuts, chips, popcorn), fast food, fatty meat |
| Sweet Energy-dense Foods | sweet pastries, jams, ice-cream, chocolate, sweets, sugar, sherbets and popsicles, sugar-sweetened beverages, desserts |
| Alcoholic Beverages | mild alcoholic beverages, liquor, liqueur, wine |
| Nuts and Seeds | unsalted nuts, almonds, seeds and peanut butter |
