# Supplementary material for: Eating Behavior Traits and Success in Dietary Changes in Men with Prediabetes – the T2D-GENE Intervention
Source: Health Educ Behav. 2026 Jan 23;53(5):440–51. doi: 10.1177/10901981251411226 (PMC13546412; doi:10.1177/10901981251411226)
Supplement: Supplementary file 5 [file sj-docx-5-heb-10.1177_10901981251411226.docx]

Supplementary Table 2

*Spearman’s Correlations Between Baseline Eating Behavior Traits and Changes in Dietary Intake ^a^ in the T2D-GENE Intervention (n=370)*

|  | **Eating behavior traits** |  |  |
| --- | --- | --- | --- |
|  | T_0_CR | T_0_UE | T_0_EE |
| **Nutrients** |  |  |  |
| Δ Energy (kcal/day) | **0.17**** | 0.06 | 0.09 |
| Δ SFA (E%) | **0.12*** | -0.10 | 0.02 |
| Δ MUFA (E%) | 0.03 | 0.02 | **0.11*** |
| Δ Fiber (g/day) | **0.11*** | **0.13*** | **0.12*** |
| **Food groups ^b^** |  |  |  |
| Δ Whole grain products | **0.12*** | 0.08 | 0.08 |
| Δ Fish | 0.01 | 0.10 | **0.11*** |
| Δ Nuts and seeds | 0.04 | 0.03 | **0.174***** |
| **Foods within the groups ^b^ and ungrouped foods** |  |  |  |
| Δ Wholegrain breads | **0.12*** | 0.07 | 0.03 |
| Δ Normal-fat milk (fat 1–1.5 %), | **0.12*** | -0.02 | 0.03 |
| Δ Fatty fermented dairy products (fat 1–4 %) | -0.04 | -0.04 | **-0.13**** |
| Δ Vegetable oil-based creams (fat 10–15 %) | 0.03 | **-0.11*** | 0.003 |
| Δ Shellfish | **0.14**** | -0.01 | 0.07 |
| Δ Processed fish products | **0.13*** | -0.05 | 0.03 |
| Δ Processed fish dishes | 0.05 | **0.19**** | 0.09 |
| Δ High-fat potato dishes | 0.03 | -0.09 | **-0.16**** |
| Δ Fatty savory pastries | **0.17**** | **-0.11*** | -0.03 |
| Δ Sugar-sweetened beverages | 0.09 | -0.01 | **-0.12*** |
| Δ Artificially sweetened beverages | -0.004 | **0.14**** | 0.02 |

Δ, delta value i.e., change from 0 to 3 years; CR, Cognitive restraint; E%, percent of daily energy intake; EE, Emotional eating; MUFA, Monounsaturated fatty acids; T_0_, baseline value; UE, Uncontrolled eating

^a^ Dietary factors that showed significant correlations with baseline eating behavior traits are presented

^b^ Food groups are presented in Supplementary table 1 (Online resource 3)

p <0.05*, p <0.01**, p<0.001***
