# Supplementary material for: Eating Behavior Traits and Success in Dietary Changes in Men with Prediabetes – the T2D-GENE Intervention
Source: Health Educ Behav. 2026 Jan 23;53(5):440–51. doi: 10.1177/10901981251411226 (PMC13546412; doi:10.1177/10901981251411226)
Supplement: Supplementary file 6 [file sj-docx-6-heb-10.1177_10901981251411226.docx]

Supplementary Table 3

*Linear Regression Analysis Between Baseline Eating Behavior Traits and Changes in Eating Behavior Traits During the T2D-GENE Intervention (n=368)*

| Change in eating  behavior traits | Predictor | R | Adjusted R^2^ | b | SE (b) | Standardized beta | t | p-value | Adjusted p-value ^a^ |
| --- | --- | --- | --- | --- | --- | --- | --- | --- | --- |
| ΔCR |  | 0.58 | 0.33 |  |  |  |  |  |  |
|  | T_0_CR |  |  | -0.56 | 0.04 | -0.57 | -13.01 | <0.001*** | <0.001*** |
|  | T_0_UE |  |  | -0.03 | 0.05 | -0.04 | -0.68 | 0.50 | 0.50 |
|  | T_0_EE |  |  | 0.16 | 0.04 | 0.21 | 4.21 | <0.001*** | <0.001*** |
| ΔUE |  | 0.54 | 0.30 |  |  |  |  |  |  |
|  | T_0_CR |  |  | -0.04 | 0.04 | -0.04 | -0.97 | 0.33 | 0.42 |
|  | T_0_UE |  |  | -0.51 | 0.04 | -0.62 | -11.93 | <0.001*** | <0.001*** |
|  | T_0_EE |  |  | 0.12 | 0.03 | 0.19 | 3.64 | <0.001*** | <0.001*** |
| ΔEE |  | 0.50 | 0.25 |  |  |  |  |  |  |
|  | T_0_CR |  |  | 0.04 | 0.05 | 0.04 | 0.89 | 0.37 | 0.42 |
|  | T_0_UE |  |  | 0.08 | 0.06 | 0.08 | 1.40 | 0.16 | 0.24 |
|  | T_0_EE |  |  | -0.42 | 0.04 | -0.53 | -10.00 | <0.001*** | <0.001*** |

Δ, delta value i.e. change from 0 to 3 years; CR, Cognitive restraint; EE, Emotional eating; T_0_, baseline value; UE Uncontrolled eating

^a^ Benjamini–Hochberg False Discovery Rate correction

p-value <0.05*; p-value <0.01**; p-value<0.001***
